# Successful splenectomy for refractory systemic lupus erythematosus–associated autoimmune hemolytic anemia: a case report and mechanistic insight

**DOI:** 10.3389/fimmu.2026.1799942

**Published:** 2026-04-28

**Authors:** Xiaoli Pan, Juan Chen, Chunyan Li, Yupei Lin, Yu Wang, Jingqiao Tian, Mei Tian, Anmao Li

**Affiliations:** Rheumatology and Immunology Department, the Affiliated Hospital of Zunyi Medical University, Zunyi, China

**Keywords:** autoimmune hemolytic anemia, IFI44L gene, splenectomy, splenic immune microenvironment, systemic lupus erythematosus, therapeutic strategies

## Abstract

Systemic lupus erythematosus (SLE) frequently involves the hematologic system, and autoimmune hemolytic anemia (AIHA) is a severe and potentially life-threatening complication. Splenectomy has been regarded as a third-line treatment option for refractory AIHA; however, its role in SLE-associated AIHA, particularly in patients with atypical immunologic phenotypes, remains to be further clarified. Here, we report a patient with refractory SLE-associated AIHA who failed multiple lines of therapy and subsequently underwent splenectomy, after which the hemoglobin level gradually increased and sustained hematologic improvement was achieved during follow-up. Splenic pathology suggested that the spleen was not only a major site of erythrocyte destruction but also a key pathologic organ in sustaining abnormal immune responses and ongoing hemolysis. In addition, positive IFI44L methylation provided supplementary supportive evidence for an SLE-related immune background. This case suggests that splenectomy may still represent a therapeutic option worthy of careful consideration in patients with refractory SLE-associated AIHA who fail multiple lines of therapy.

## Introduction

Autoimmune hemolytic anemia (AIHA) is an acquired disorder characterized by immune-mediated destruction of red blood cells, resulting in hemolytic decompensation (1). Its annual incidence is estimated at 1–3 cases per 100,000 persons, with a mortality rate of approximately 11% (2). Systemic lupus erythematosus (SLE), a multisystem autoimmune disease, may be complicated by AIHA, with a reported prevalence of approximately 5%–10% (3). Compared with primary AIHA, SLE-associated AIHA is often more complex and less predictably responsive to treatment. At present, first-line therapy for AIHA remains based on glucocorticoids and immunosuppressive agents, whereas second-line treatments such as rituximab (RTX) are generally required in refractory cases. Splenectomy has been regarded as a third-line treatment option for refractory AIHA; however, its role in SLE-associated AIHA, particularly in patients with atypical immunologic features, remains to be further clarified.

Here, we report a patient with refractory SLE-associated AIHA who had received multiple treatment modalities, including high-dose glucocorticoids, mycophenolate mofetil (MMF), cyclosporine (CAs), RTX, intravenous immunoglobulin (IVIG), and therapeutic plasma exchange (TPE), with unsatisfactory responses. Sustained hematologic improvement was ultimately achieved after splenectomy. In conjunction with splenic pathology and interferon-induced protein 44-like (IFI44L) methylation findings, we further explored the potential role of the spleen in such refractory cases and its clinical significance.

## Case report

The patient was a 44-year-old woman who presented to the hospital in August 2024 with a 3-day history of fatigue, poor appetite, and epigastric discomfort. During the disease course, she denied alopecia, photosensitivity, oral ulcers, arthralgia, or involvement of the skin, kidneys, or nervous system. Laboratory investigations revealed the following: White blood cell count: 2.74 × 10^9^/L (reference range: 3.5-9.5×10^9^/L); Hemoglobin: 41 g/L (reference range: 115–150 g/L); Absolute reticulocyte count: 228.7 × 10^9^/L (reference range: 25.7-75.0×10^9^/L); Reticulocyte percentage: 14.6% (reference range: 0.5-1.5%); Lactate dehydrogenase (LDH): 681 U/L (reference range: 140–271 U/L); Total bilirubin: 49.5 μmol/L (reference range: 5-21 μmol/L); Indirect bilirubin: 39.2 μmol/L (reference range: 1.7–13.7 μmol/L); D-dimer: 26.49 μg/mL (reference range: <1.0 μg/mL); C-reactive protein (CRP): 23.94 mg/L (reference range: 0.068-8.2 mg/L); Rheumatoid factor: 459 U/mL (reference range: <20 U/mL); Immunoglobulin G (IgG): 4.2 g/L (reference range: 7.51-15.6 g/L); Complement C3: 0.63 g/L (reference range: 0.79-1.52 g/L); Complement C4: < 0.02 g/L (reference range: 0.16-0.38 g/L); Antinuclear antibody (ANA): Weakly positive (titer 1:100); Ferritin: 369.0 μg/L (reference range: 11-306.8μg/L); Urine hemosiderin qualitative test: negative. Direct antiglobulin test (DAT): Positive (titer 1:16); Lupus anticoagulant screen: LA1 55.8 seconds, LA2 29.6 seconds, Normalized Ratio 1.89 (negative range: 0.8-1.19); Anticardiolipin antibody: Positive; All quantitative anticardiolipin antibody tests (aCL-IgA, IgG, and IgM) and anti-β2 glycoprotein I antibody tests (β2-GP1 IgA, IgG, and IgM) were within the normal range. Antinuclear antibody (ANA) profile: Unremarkable. Imaging revealed splenomegaly with splenic vein dilation (max diameter ~17 mm) and chest CT demonstrated left lower lobe bronchiectasis with infection, while initial lower extremity Doppler ultrasound showed no thrombosis. Bone marrow biopsy confirmed erythroid hyperplasia without dysplastic changes in erythroid precursors or mature erythrocytes. The preliminary diagnosis was AIHA, suspected to be secondary to SLE.

Initial therapy with intravenous dexamethasone 10 mg daily resulted in hemoglobin elevation to 95 g/L. Upon discharge, the regimen was transitioned to oral prednisone 60 mg daily combined with MMF 0.5 g twice daily. In November 2024, the patient re-presented with fatigue and was found to have hemoglobin decreased to 39 g/L with positive red blood cell autoantibodies. Diagnostic evaluations revealed normal ADAMTS13 activity and negative paroxysmal nocturnal hemoglobinuria (PNH) testing. PET-CT showed no evidence of malignancy, while Doppler ultrasound identified a right calf muscular vein thrombosis. Serial monitoring demonstrated progressive normalization of antinuclear antibody, anticardiolipin antibody, and lupus anticoagulant ratios. Subsequent intervention comprised intravenous methylprednisolone pulse therapy (200 mg daily for 5 days), concurrent IVIG 20 g daily for 3 days, therapeutic anticoagulation with rivaroxaban 20 mg daily, and supportive packed red blood cell transfusions, achieving post-treatment stabilization with white blood cell count 2.84 × 10^9^/L and hemoglobin 70 g/L, culminating in a discharge regimen of prednisone 50 mg daily plus MMF 0.75 g twice daily.

**Figure 1:**
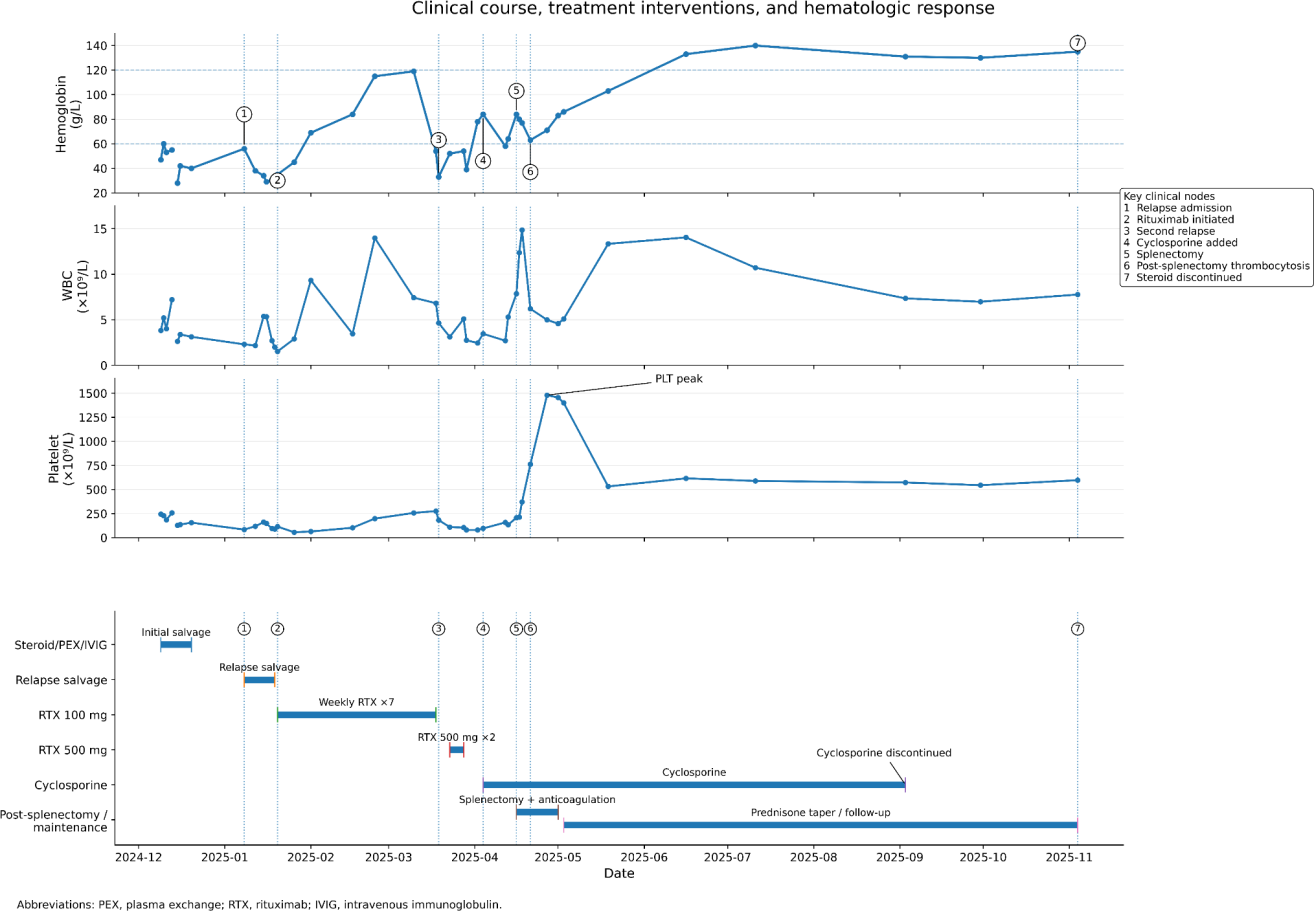
Clinical course, treatment interventions, and hematologic response of the patient. The upper three panels show serial changes in hemoglobin (Hb), white blood cell count (WBC), and platelet count (PLT) during follow-up, while the lower timeline summarizes the major treatment interventions and key clinical nodes. Following splenectomy, the patient’s hemoglobin level gradually increased, with sustained hematologic improvement during subsequent follow-up. PEX, plasma exchange; RTX, rituximab; IVIG, intravenous immunoglobulin.

**Figure 2:**
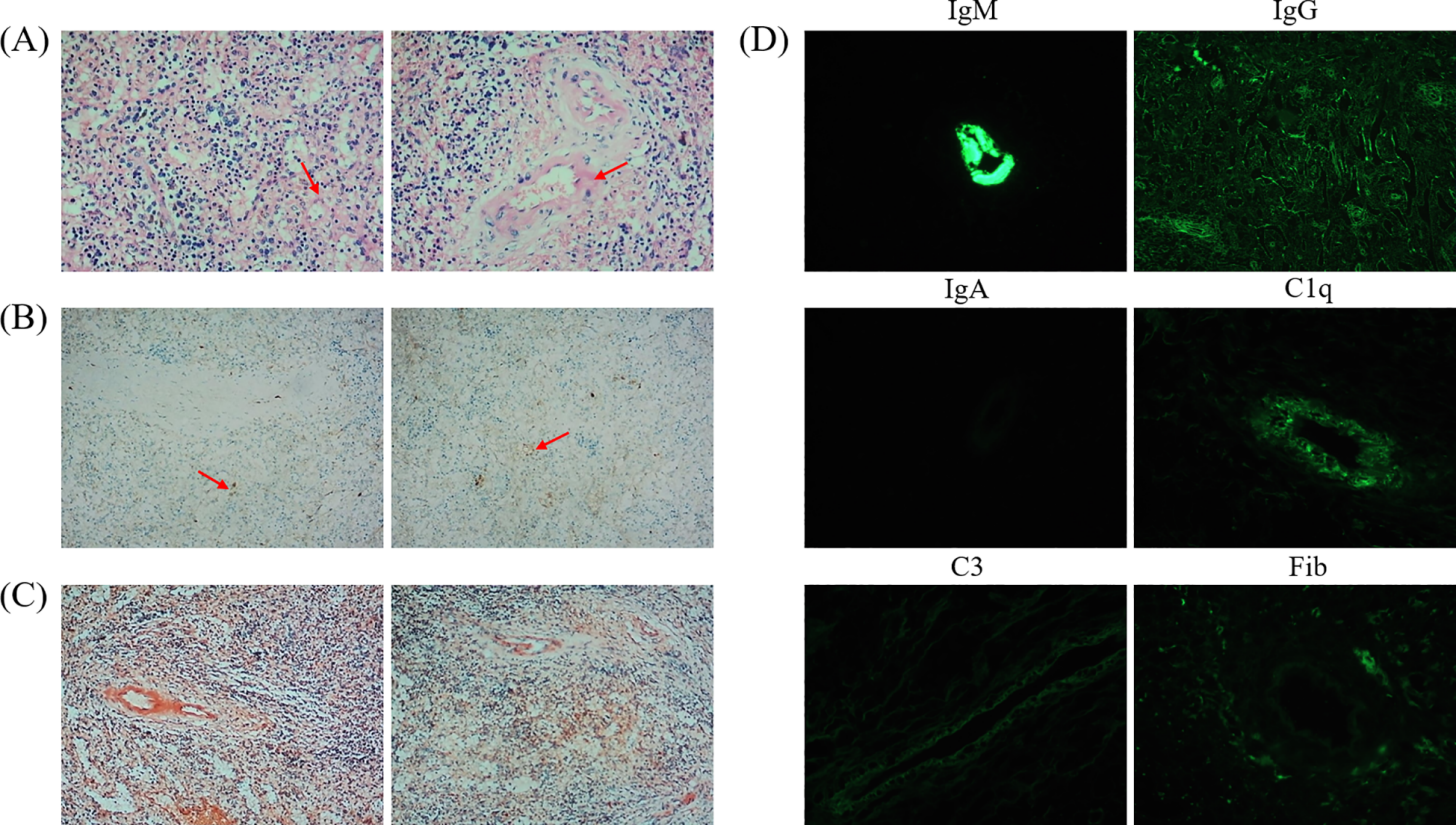
Splenic histopathological findings. **(A)** Hematoxylin and eosin (H&E) staining demonstrates preserved splenic architecture with dilated sinusoids. Trilineage hematopoietic cells are observed. Select arterioles exhibit mural thickening containing amorphous eosinophilic deposits (red arrows). **(B)** Immunohistochemistry reveals focal kappa light chain-restricted plasma cells (left) and lambda light chain-restricted plasma cells (right) (red arrows). **(C)** Congo red (left) and oxidized Congo red (right) staining show no amyloid deposition within vascular walls. **(D)** Immunofluorescence demonstrates vascular wall positivity: IgM (+++), IgG (+), IgA (-), C1q (+), C3 (focal/weak positivity), fibrinogen (-).

In December 2024, disease recurrence manifested with hemoglobin at 47 g/L (supporting laboratory data in **Table 1**). Therapy comprised intravenous methylprednisolone 500 mg daily for 3 days, three sessions of TPE, and IVIG 20 g daily for 3 days, supplemented with intermittent leukocyte-reduced red blood cell transfusions (total 10 units). Post-treatment hemoglobin remained at 40 g/L. Following referral to Southwest Hospital Chongqing, the patient received intravenous methylprednisolone 40 mg daily plus IVIG 20 g daily for 6 days with suboptimal response. Upon readmission to our institution in January 2025 (hemoglobin 56.0 g/L), sequential therapies including dexamethasone, IVIG, and TPE were reinitiated without clinical improvement. Subsequent treatment with RTX 100 mg weekly for 8 weeks elevated hemoglobin to 119 g/L; however, rapid recurrence occurred eight days later (hemoglobin 54 g/L). A second RTX course (500 mg weekly × 2 doses) proved ineffective, prompting intravenous methylprednisolone 250 mg daily for 3 days combined with oral cyclosporine 50 mg twice daily and supportive transfusions. Despite these interventions, refractory hemolysis persisted, necessitating splenectomy by hepatobiliary surgery. At the last follow-up on November 4, 2025, the patient’s complete blood count showed a white blood cell count of 7.77 × 10^9^/L, a hemoglobin level of 135 g/L, and a platelet count of 597 × 10^9^/L. The total bilirubin level was 12.8 μmol/L, indirect bilirubin was 2.4 μmol/L, and LDH was 260 U/L. Postoperative hemoglobin demonstrated progressive recovery (**Table 1**; **Figure 1**), with splenic histopathology detailed in **Figure 2**. Further serum immunofixation electrophoresis revealed no abnormal protein bands. Because the patient exhibited some SLE-related clinical and immunologic features but lacked typical SLE-specific autoantibodies, and because the conventional immunologic findings were insufficient to fully explain the complexity of the case, IFI44L methylation testing related to systemic lupus erythematosus was further performed using the PCR melting curve method. The result was positive (19%; negative reference threshold, >25%). This finding was interpreted as supplementary supportive evidence for an SLE-related immune background.

**Table 1:** Laboratory results and therapeutic course.

Date	Hb (g/L)	WBC (10^9^/L)	PLT (10^9^/L)	Transfusion volume (U)	Interventions and clinical events
2024-12-09	47	3.82	244	2	Methylprednisolone 80 mg every 12 hours
2024-12-10	60	5.21	228	–	Methylprednisolone 500 mg daily (×3 days)
2024-12-11	53	4.01	185	–	TPE (×3 sessions)
2024-12-13	55	7.21	258	–	Methylprednisolone 80 mg daily
2024-12-14	–	–	–	–	IVIG 20 g
2024-12-15	28	2.63	128	4	IVIG 20 g
2024-12-16	42	3.39	136	2	IVIG 20 g
2025-01-08	56	2.3	84	2	Readmitted with recurrent hemolysis; Dexamethasone 10 mg daily
2025-01-12	38	2.17	118	1	Dexamethasone 20 mg daily
2025-.01-15	34	5.37	162	2	TPE resumed (×3 sessions)
2025-01-18	31	2.7	95	1	IVIG 22.5 g daily (×3 days)
2025-01-20	35	1.52	115	2	**RTX 100 mg (Cycle 1)**
2025-01-26	45	2.9	56	2	**RTX 100 mg (Cycle 2)**
2025-02-01	69	9.33	64	–	**RTX 100 mg (Cycle 3)**
2025-02-16	84	3.46	103	–	**RTX 100 mg (Cycle 4);** Prednisone 50 mg daily initiated
2025-02-24	115	13.98	198	–	**RTX 100 mg (Cycle 5);** Prednisone tapered to 40 mg daily
2025-03-10	119	7.43	257	–	**RTX 100 mg (Cycle 6);** Prednisone tapered to 30 mg daily
2025-03-18	54	6.82	276	–	**RTX 100 mg (Cycle 7);** Prednisone tapered to 10 mg daily
2025-03-19	33	4.64	182	2	Readmitted with recurrent hemolysis; Dexamethasone 10 mg daily
2025-03-23	52	3.12	110	2	**RTX 500 mg (Cycle 1)**
2025-03-28	54	5.09	105	2	**RTX 500 mg (Cycle 2)**
2025-03-29	39	2.75	80	2	Methylprednisolone 250 mg daily (×3 days)
2025-04-04	84	3.46	96	–	Cyclosporine 50 mg twice daily added
2025-04-12	58	2.70	158	2	Hepatobiliary surgery consultation
2025-04-13	64	5.30	134	2	
2025-04-16	84	7.87	207	–	Transferred for splenectomy
2025-04-17	80	12.37	213	–	Postoperative day 1: Dexamethasone 10 mg daily
2025-04-18	77	14.86	371	–	Postoperative day 2
2025-04-21	63	6.21	762	–	Marked thrombocytosis post-splenectomy; Dalteparin anticoagulation initiated
2025-04-27	71	4.99	1480	–	Persistent thrombocytosis
2025-05-01	83	4.57	1455	–	Dalteparin discontinued; Rivaroxaban 20 mg daily initiated
2025-05-03	86	5.09	1400	–	Maintenance: Prednisone 40 mg daily + Cyclosporine 50 mg twice daily + Rivaroxaban 20 mg daily
2025-06-16	133	14.05	616	–	Prednisone tapered to 30 mg daily
2025-07-11	140	10.72	589	–	Prednisone tapered to 20 mg daily
2025-09-03	131	7.35	573	–	Prednisone tapered to 10 mg daily、Cyclosporine discontinued
2025-09-30	130	6.98	545	–	
2025-11-04	135	7.77	597	–	Glucocorticoids discontinued

Bolded entries indicate the main time points and doses of RTX administration for the patient.

## Discussion

AIHA is closely associated with the loss of immune tolerance to erythrocyte self-antigens and aberrant activation of the complement system. It represents a relatively uncommon but potentially life-threatening hematologic manifestation of systemic lupus erythematosus (SLE). Compared with primary AIHA, SLE-associated AIHA is often more refractory and severe, characterized by greater heterogeneity of circulating autoantibodies and more complex systemic involvement (4, 5). In the present case, the patient initially presented with severe hemolytic anemia accompanied by marked splenomegaly, fulfilling the diagnostic criteria of the 2023 Chinese guidelines for adult autoimmune hemolytic anemia. In addition, the patient exhibited leukopenia, hypocomplementemia, weakly positive antinuclear antibodies, partial positivity for autoantibodies, and immune-mediated hemolysis in the early stage, collectively suggesting an SLE-related immunologic background. Although the patient lacked typical SLE-specific autoantibodies and some autoantibodies became negative during follow-up, based on the 2012 SLICC criteria, the 2019 EULAR/ACR classification criteria, and the 2023 updated EULAR recommendations for the management of SLE, together with the positive IFI44L gene methylation result and pathological findings demonstrating local complement activation, IgM-dominant immune complex deposition, extramedullary hematopoiesis, and plasma cell infiltration in the spleen, we consider this case to be more consistent with atypical hematologic involvement in the context of an SLE-related immune background (6–9), rather than primary AIHA. During the disease course, the patient received multiple lines of therapy, including high-dose glucocorticoids, MMF, CAs, RTX, IVIG, and TPE. However, the patient continued to experience recurrent episodes of severe hemolysis and remained transfusion-dependent for a prolonged period. Although a transient increase in hemoglobin was observed following rituximab therapy, relapse occurred shortly thereafter, indicating that conventional immunosuppressive therapy and B-cell depletion were insufficient to achieve sustained disease control. This therapeutic failure constituted the key clinical rationale for considering splenectomy.

The spleen is not only the principal site for the clearance of antibody-coated erythrocytes but also an important organ in which abnormal humoral immune responses may persist. In SLE-associated AIHA, the spleen functions as a central hub of systemic immune dysregulation, and its nonhematopoietic stromal cell network may exert important immunoregulatory effects. Acetylcholine (ACh) derived from splenic fibroblastic reticular cells (FRCs) can promote mitochondrial reactive oxygen species (ROS) generation, thereby continuously amplifying the activation and proliferation of autoreactive B cells and driving the production of anti-erythrocyte autoantibodies (10). In refractory cases, CD19^-^BCMA^+^ long-lived plasma cells (LLPCs) frequently accumulate in the spleen and serve as a major source of autoantibodies (11, 12). These cells may continuously drive the pathogenic process of AIHA and are resistant to conventional B-cell–depleting therapies such as RTX. In addition, the accumulation of spleen-derived CD11b^+^Gr-1^+^ myeloid cells (SDMCs) may further promote LLPCs expansion and antibody production (13). Moreover, overactivation of splenic macrophages may enhance erythrophagocytosis and directly mediate hemolytic anemia (14). In the present case, the patient had marked splenomegaly, and splenic pathology further demonstrated extramedullary hematopoiesis, plasma cell infiltration, predominant IgM deposition, and complement-related abnormalities. These findings further suggest that the splenic immune microenvironment may play a central role in refractory SLE-associated AIHA, and that these diverse pathological alterations may form an interactive network that sustains and amplifies hemolysis. We therefore speculate that, in this patient, the spleen served a dual role as both a “site of erythrocyte destruction” and an “organ maintaining abnormal immune responses,” thereby providing a pathophysiologic basis for the efficacy of splenectomy.

Of note, splenic immunofluorescence in this patient demonstrated strong IgM positivity (+++), C1q positivity (+), and weak/partial C3 positivity (+/−), whereas IgG staining was relatively weak. This pattern suggests that the immunophenotype was not entirely consistent with typical warm antibody AIHA, but instead showed features of cold antibody predominance and/or complement involvement (15). According to current understanding, splenectomy is generally more suitable for warm AIHA, whereas its efficacy in typical cold AIHA is often limited (16, 17). Therefore, the distinctive feature of this case lies in the fact that, despite the IgM/complement-dominant pattern, the patient still achieved sustained improvement after splenectomy. We believe this may be explained by the fact that this case did not represent a typical pure cold agglutinin disease, but rather a complex disorder characterized by marked splenomegaly, local immune deposition, plasma cell infiltration, and extramedullary hematopoiesis. Under these circumstances, the spleen may have played a far more central role in ongoing disease activity than in typical cold AIHA, thereby allowing splenectomy to remain effective.

The decision to perform splenectomy in this patient was not based solely on empirical salvage therapy, but rather on multiple considerations, including failure of several lines of medical treatment, persistent severe hemolysis, marked splenomegaly, and the likelihood that the spleen served as a key pathologic organ. In the early postoperative period, the patient continued to receive glucocorticoids, which were gradually tapered thereafter. Serial monitoring showed a progressive increase in hemoglobin levels, and sustained hematologic improvement was observed during follow-up. These findings suggest that, in this case, a splenectomy-centered combined therapeutic approach played a key role in disease control.

IFI44L is an immune-related gene that belongs to the interferon-stimulated gene (ISG) family and participates in the type I interferon (IFN-I) signaling pathway. DNA hypomethylation in its promoter region has been closely associated with abnormal CD4^+^ T-cell activation and disease progression in SLE (6, 18). Overactivation of the IFN pathway is a key pathogenic mechanism in SLE, and approximately 50%–75% of patients with SLE exhibit upregulated expression of IFN-regulated genes (19). In the present case, positive IFI44L methylation testing provided additional supportive evidence for an SLE-related immune background (6). A positive IFI44L result suggests possible abnormal activation of the IFN-I pathway and may reflect deeper SLE-related immune dysregulation. Previous studies have suggested that IFN-I can upregulate complement components such as C1q and C3, activate macrophages, suppress erythropoiesis, and promote B-cell differentiation into plasma cells (20–22). However, the precise causal relationship between IFN-I pathway activation and persistent hemolysis, local splenic pathological changes, and treatment resistance has not yet been established and warrants further investigation.

The success of splenectomy in this patient may be explained by the fact that it simultaneously removed the principal site of erythrocyte destruction, eliminated long-lived plasma cells that continuously produced autoantibodies, and interrupted the complement-mediated local inflammatory cycle, thereby breaking the vicious cycle of refractory AIHA. This case suggests that splenectomy may still represent a therapeutic option worthy of careful consideration in patients with refractory SLE-associated AIHA who exhibit recurrent severe hemolysis, failure of multiple lines of immunosuppressive therapy, marked splenomegaly, and pathological evidence of substantial splenic involvement. Even in patients with certain IgM- or complement-dominant features, splenectomy should not necessarily be considered ineffective if the spleen occupies a central role in disease pathogenesis. In the future, precision therapies targeting complement, plasma cells, or interferon pathways may provide additional options for such patients; however, in current clinical practice, splenectomy still retains practical and important therapeutic value in selected refractory cases.

## Conclusion

The development and progression of SLE-associated AIHA may involve the combined effects of systemic immune dysregulation and spleen-centered local pathologic alterations. This case suggests that the spleen may serve not only as a major site for the clearance of antibody-coated erythrocytes but also as a key organ in maintaining abnormal immune responses and ongoing hemolysis. The marked clinical improvement achieved after splenectomy in this patient further supports the pivotal role of the spleen in sustaining the vicious cycle of autoimmunity and hemolysis. For patients with SLE-associated AIHA who share similar refractory features, splenectomy may still represent a therapeutic option worthy of careful consideration. In addition, treatment strategies may need to shift toward a multi-targeted therapeutic paradigm. Further cases and studies are needed to better define which patients are most likely to benefit from this approach.

## Data Availability

The original contributions presented in the study are included in the article/supplementary material. Further inquiries can be directed to the corresponding authors.

## References

[1] Barcellini W . Immune hemolysis: Diagnosis and treatment recommendations. Semin Hematol. (2015) 52:304–12. doi: 10.1053/j.seminhematol.2015.05.001. PMID: 26404442

[2] Harahap RIM Ardiningrum T Pamela Y Widiasta A Rossanti R Bethasari M . Prevalence of systemic lupus erythematosus in autoimmune hemolytic anemia patients based on coombs test results. Eur J Med Res. (2025) 30:351. doi: 10.1186/s40001-025-02601-8. PMID: 40312681 PMC12046922

[3] Domiciano DS Shinjo SK . Autoimmune hemolytic anemia in systemic lupus erythematosus: Association with thrombocytopenia. Clin Rheumatol. (2010) 29:1427–31. doi: 10.1007/s10067-010-1479-2. PMID: 20473539

[4] Fayyaz A Igoe A Kurien BT Danda D James JA Stafford HA . Haematological manifestations of lupus. Lupus Sci Med. (2015) 2:e000078. doi: 10.1136/lupus-2014-000078. PMID: 25861458 PMC4378375

[5] Hill QA Stamps R Massey E Grainger JD Provan D Hill A . Guidelines on the management of drug-induced immune and secondary autoimmune, haemolytic anaemia. Br J Haematol. (2017) 177:208–20. doi: 10.1111/bjh.14654. PMID: 28369704

[6] Zhao M Zhou Y Zhu B Wan M Jiang T Tan Q . IFI44L promoter methylation as a blood biomarker for systemic lupus erythematosus. Ann Rheum Dis. (2016) 75:1998–2006. doi: 10.1136/annrheumdis-2015-208410. PMID: 26787370 PMC4955646

[7] Dijkstra DJ van de Bovenkamp FS Abendstein L Zuijderduijn R Pool J Kramer CSM . Human anti-C1q autoantibodies bind specifically to solid-phase C1q and enhance phagocytosis but not complement activation. Proc Natl Acad Sci U S A. (2023) 120:e2310666120. doi: 10.1073/pnas.2310666120. PMID: 38048459 PMC10723154

[8] Bernardoff I Picq A Loiseau P Foret T Dufrost V Moulinet T . Antiphospholipid antibodies and the risk of autoimmune hemolytic anemia in patients with systemic lupus erythematosus: A systematic review and meta-analysis. Autoimmun Rev. (2022) 21:102913. doi: 10.1016/j.autrev.2021.102913. PMID: 34371159

[9] Kleer JS Rabatscher PA Weiss J Leonardi J Vogt SB Kieninger-Gräfitsch A . Epitope-specific anti-C1q autoantibodies in systemic lupus erythematosus. Front Immunol. (2022) 12:761395. doi: 10.3389/fimmu.2021.761395. PMID: . Published 2022 Jan 11. 35087514 PMC8788646

[10] Zeng Q Wang S Li M Wang S Guo C Ruan X . Spleen fibroblastic reticular cell-derived acetylcholine promotes lipid metabolism to drive autoreactive B cell responses. Cell Metab. (2023) 35:837–54.e8. doi: 10.1016/j.cmet.2023.03.010. PMID: 37019104

[11] Feng J Huo D Hong R Jin X Cao H Shao M . Co-infusion of CD19-targeting and BCMA-targeting CAR-T cells for treatment-refractory systemic lupus erythematosus: a phase 1 trial. Nat Med. (2025) 31:3725–36. doi: 10.1038/s41591-025-03937-8. PMID: 40993243

[12] Doglio M Alexander T Del Papa N Snowden JA Greco R Autoimmune Diseases Working Party (ADWP) of the European Society for Blood and Marrow Transplantation (EBMT) . New insights in systemic lupus erythematosus: From regulatory T cells to CAR-T-cell strategies. J Allergy Clin Immunol. (2022) 150:1289–301. doi: 10.1016/j.jaci.2022.08.003. PMID: 36137815

[13] Jang E Cho S Pyo S Nam JW Youn J . An inflammatory loop between spleen-derived myeloid cells and CD4 T cells leads to accumulation of long-lived plasma cells that exacerbates lupus autoimmunity. Front Immunol. (2021) 12:631472. doi: 10.3389/fimmu.2021.631472. PMID: 33643317 PMC7904883

[14] Guo H Zhang H Sheng N Wang J Chen J Dai J . Perfluorooctanoic acid (PFOA) exposure induces splenic atrophy via overactivation of macrophages in male mice. J Hazard Mater. (2021) 407:124862. doi: 10.1016/j.jhazmat.2020.124862. PMID: 33360190

[15] Jäger U Barcellini W Broome CM Gertz MA Hill A Hill QA . Diagnosis and treatment of autoimmune hemolytic anemia in adults: Recommendations from the first international consensus meeting. Blood Rev. (2020) 41:100648. doi: 10.1016/j.blre.2019.100648, PMID: 31839434

[16] Hill QA Stamps R Massey E . The diagnosis and management of primary autoimmune haemolytic anaemia. Br J Haematol. (2017) 176:395–411. doi: 10.1111/bjh.14478. PMID: 28005293

[17] Berentsen S Fattizzo B Barcellini W . The choice of new treatments in autoimmune hemolytic anemia: How to pick from the basket? Front Immunol. (2023) 14:1180509. doi: 10.3389/fimmu.2023.1180509. PMID: 37168855 PMC10165002

[18] Hiramatsu S Watanabe KS Zeggar S . Regulation of Cathepsin E gene expression by the transcription factor Kaiso in MRL/lpr mice derived CD4+ T cells. Sci Rep. (2019) 9:3054. doi: 10.1038/s41598-019-38809-y. PMID: 30816218 PMC6395770

[19] Postal M Vivaldo JF Fernandez-Ruiz R Paredes JL Appenzeller S Niewold TB . Type I interferon in the pathogenesis of systemic lupus erythematosus. Curr Opin Immunol. (2020) 67:87–94. doi: 10.1016/j.coi.2020.10.014. PMID: 33246136 PMC8054829

[20] Liu Y Pal M Bao W . Type I interferon is induced by hemolysis and drives antibody-mediated erythrophagocytosis in sickle cell disease. Blood. (2021) 138:1162–71. doi: 10.1182/blood.2021011629. PMID: 34166491 PMC8570059

[21] Morel L . Erythrocyte-derived mitochondria: An unexpected interferon inducer in lupus. Trends Immunol. (2021) 42:1054–6. doi: 10.1016/j.it.2021.10.010. PMID: 34764015

[22] Fillatreau S . Antibodies against type I IFN: The bad guys self-restrain in systemic lupus erythematosus. Cell Rep Med. (2023) 4:100903. doi: 10.1016/j.xcrm.2022.100903. PMID: 36652912 PMC9873922

